# Urban Growth-Oriented Green Accumulation: Ecological Conservation Planning in the Shenzhen DaPeng Peninsula in Southern China

**DOI:** 10.3390/ijerph16010104

**Published:** 2019-01-02

**Authors:** Dan Lin

**Affiliations:** Department of Tourism Management, Shenzhen Tourism College, Jinan University, 518053 Shenzhen, China; lindan@sz.jnu.edu.cn

**Keywords:** neoliberal conservation, urban growth, green grabbing, local peasants, DaPeng Peninsula, China

## Abstract

Neoliberal conservation has recently become a topic of academic research and a method of practice within the context of globalization. Less attention has been given to how neoliberal conservation has been practiced at the urban scale. This paper draws on the concept of ‘urban-growth-oriented green grabbing’ to capture the multidimensionality of the reasoning and process of ecological conservation in an urban growth context. It focuses on two ecological spatial protection plans for the DaPeng Peninsula in the city of Shenzhen, China. Through empirical investigation, this article traces the political economy of these plans and draws out the insights they offer regarding theories of urban environmentalism. The empirical results show that the ecological conservation plans are best understood as ‘green grabbing’ that has been achieved by land transfer and spatial interest redistribution. Conceptually, the paper provides further evidence of the process of neoliberal conservation within the urban context.

## 1. Introduction

The rapid expansion of conservation territories since 1990s has been described by Zimmerer as a reworking of capitalist modernity [1]. The parallel processes of globalization-renationalization and commodification-privatization continue to contribute to the profusion of geographical production of conservation, leading to a ‘new enclosures movement’ [1]. This wave of conservation territories covers a range of planning and schemes, including parks-with-people projects, conservation-with-development or wildlife utilization projects [2], the Integrated Conservation Development project, Wetland mitigation banking to create a functioning market in ecosystem services [3], and the development of stable markets in ecosystem services [4]. What is emerging in China is a set of practices which has focused its energies and resources primarily on ecological management, involving the creation of proposing, assessing and ratifying conservation areas at various levels of government [5,6,7]. Within the strengthened ecological regulation regime, according to the MEP [8] the number of nature reserves increased to 2750 by 2016, with their total area of over 1.47 million km^2^ amounting to 14.9% of China’s territory.

For those scholars taking the perspective of political economy, conservation territorialization constitutes one of the most important dimensions of the current era of neoliberalization of nature [9] which highlights the increasing importance of economic and market dynamics in ecological reform and the increased adoption of environmental policy objectives by private business [10]. The core of these discussions has been the exploration of the relationship between capital and nature in the context of globalization [11], the fundamental reasoning of neo-liberal conservation [12], and the process of ‘accumulation through dispossession’ [13,14]. Recent research starts to propose a synthesis between nature and cities, which leads to seeing urban growth as the main context of and sometimes the impetus for ecological conservation within the wave of conservation territorialization [15,16,17]. More empirical studies need to be done on investigating how conservation has been practiced at the urban scale and what implications this might have for the politics and practices of urban and regional development and conservation in itself.

The following paper relocates ecological conservation schemes within the context of socioeconomic and political transition in urban China and explores two conservation plans in the DaPeng Peninsula (henceforth the DP) in Shenzhen. As the first special economic zone located in the Pearl River Delta region, Shenzhen has been held up as a prime example of rapid economic growth and urbanization in China under reform and openness programs since 1978. Shenzhen’s local political economy after 1978 had been highly embedded in the context of securing growth and investment. Located southeast of Shenzhen city, the DP is the largest ecologically sensitive area preserved in the city. The region is 296 km^2^ with a total coastline of 133.22 km. Since 2003, stringent ecological regulatory strategies have been promoted on the DP, which are represented by the Shenzhen Eastern Ecological Group Plan (2005–2020) (the Plan) [18]. This plan has established the requirements for ecological regulation in the DP. In 2007, the DaPeng Peninsula Protection and Development Implementation Strategy (the Strategy) [19] was promulgated, which is a further attempt by the municipal government to deregulate the peninsula, employing a concept of ‘graded protection areas’ to govern land on the DaPeng Peninsula based on the ideas of ‘core ecological protection zones’ (CEPZ) and ‘construction control areas’ (CCA).

This paper’s objectives are threefold: (1) to theorize suburban nature conservation in China through an examination of the formulation and implementation process of two conservation plans in the DP; (2) to reflect on what the case study reveals about the logic of ecological protection in the context of China’s urban political economy; and (3) to discuss more broadly on how the Chinese case contributes to the theory of urban environmentalism. The main research questions of this article are: why and how ‘wild’ or ‘natural’ areas are captured by and incorporated into urban space, and how land appropriation for urban ecological amenities forms a new model of appropriation in China?

## 2. Suburban Nature Conservation: Urban-Growth-Oriented Green Grabbing

Neoliberalism denotes a complex assemblage of ideological commitments, discursive representations, and institutional practices that involve a wide range of actors at multiple geographical scales [20]. Within this discourse, a growing literature explores the implications of neoliberalism for biodiversity conservation and its resulting ‘neoliberalization of nature’ [11,12,21]. Neo-liberal approaches to ecological conservation are approaches that focus ‘on economic benefits and costs of biodiversity erosion and management. It emphasizes the central role of the market in regulating the use of natural resources and a more limited role for the state, which retreats from intervention to fulfil the roles of standard setting and “refereeing” the proper functioning of markets’ [22].

A growing body of literature explores the implications of neoliberalism for nature conservation and its resulting neoliberalization of nature [11,12,21]. Neoliberalism denotes a complex assemblage of ideological commitments, discursive representations, and institutional practices that involve a wide range of actors at multiple geographical scales [20]. Neo-liberal approaches to ecological conservation are approaches that focus ‘on economic benefits and cost of biodiversity erosion and management. It emphasizes the central role of the market in regulating the use of natural resources and a more limited role for the state, which retreats from intervention to fulfil the roles of standard setting and ‘refereeing’ the proper functioning of markets’ [22]. The widely accepted rationale for neoliberalizing nature starts from ‘the double movement’ concept [23]. As argued by O’Connor [24], nature is the foundation on which capitalist firms depend for wealth accumulation. The extensive processing of environmental resources may lead to crises of economic profitability as environmental problems proliferate and become costly. The economy-environment contradictions that Polanyi, O’Connor, and other critics identify can be ameliorated and even overcome not by fencing off the nonhuman world (e.g., through state protection) but by bringing it fully within the universe of capital accumulation.

Under the assumption of urban-rural dualism, and followed the tradition of fortress conservation [25], cities are perceived to exist outside wild nature; therefore, most studies of neoliberal conservation are executed in rural areas far from the city. Within the context that urbanization has increasingly affected the functioning of protected areas [26], recent studies, however, have linked ecological conservation with urban to investigate why and how nature conservation works within urban systems [27]. These research focus mainly on three aspects: (1) exploring the political background where ecological conservation policies are formulated [28,29], (2) understanding the role of ecological conservation policy to entrepreneurial urban development strategies [30,31], (3) analyzing the impact of ecological conservation within urban context (e.g., [32]).

**Figure 1:**
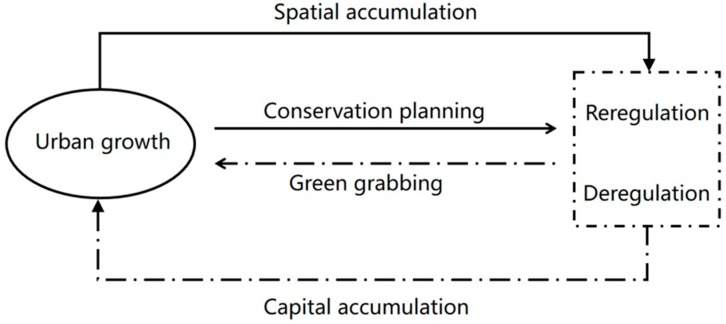
The Analytical Framework.

The exploration of urbanity in neo-liberalizing nature requires further research [33]. Studies on urban environmentalism are mainly based on urban environment yet pay less attention to conservation in rural areas. In the following section, I construct a theoretical analysis framework by the combination of the theory of neoliberalising nature and urban governance theory to help conceptualize ecological conservation within urban planning and implementation. I argue that the planning and implementation of ecological conservation within cities can be regarded as the way of ‘urban-growth-oriented green grabbing’ (see Figure 1). A set of propositions based on theoretical discussions is advanced to elaborate the analytical framework.

### The Analytical Framework: Urban-Growth-Oriented Green Grabbing

**Proposition 1** **.** *Within the context of economic globalization, the main objective of urban governance is to achieve urban growth. The designation of nature reserves supports cities in enhancing city competitiveness, and thus towards the aim of urban growth.*

Molotch [34] notes that the very essence of a locality is its operation as a growth machine, where the desire for growth provides the key operative motivation towards consensus for private and public stakeholders in relation to land development. Harvey [35] goes further and proposes the argument that the city could play a role as an active agent to pursue entrepreneurial advantages as an enterprise. Cities with entrepreneurialism could be defined as strategic actors, who, through competitive strategies, proactive approaches and innovative images, build geographically based strategies to enhance the cities’ structural competitiveness to fix the eternally migrating capital within the context of economic globalisation [36].

Drawing on Harvey’s concept of spatial fixes, while Jonas and Gibbs argue that pressures and demands for more stringent environmental and ecological regulation create problems and opportunities for urban growth regimes in a context where cities compete to attract and hold down investment [37,38]. For certain types of city, there are powerful competitive pressures to become clean and green as part of the urban consumption amenities needed to secure high-road economic growth. The rationale on conservation within urban context, therefore, has been described by While et al. [39], p. 551) as ‘urban sustainability fixes’. Lin further brings in the term ‘biophysical fixes’ and applies it in the urban context to represent a subset of ‘sustainability fixes’, which demonstrates how ecological conservation schemes have been integral parts of many cities where entrepreneurial urban development strategies are practiced under conditions of climate change and resource constraint [31]. These studies note that city competitiveness lies in the capability of cities to secure scarce natural resources to maintain and enhance economic growth within the greening of governance. In such context, contributions that ecological conservation may provide are twofold. On one hand, opportunities for cities and regions to engage in ecological conservation markets to offset higher financial inputs for conservation [3,4,39]. On the other hand, ecological conservation becomes necessary to a continued supply of resources for the city’s future economic growth, because conservation territories may break the land provision limits in the future [16].

**Proposition 2** **.** *The transformation from space accumulation to capital accumulation is achieved through the process of green grabbing. That is, to convert the land ownership and use rights of protected areas that are not originally cities into urban governments through the designation of nature reserves; and to redefine methods of ecological resources development through further territorialization.*

According to Harvey, the designation of nature reserves comprise an important dynamic of ‘accumulation through dispossession’ [14]. This concept theorizes the on-going processes of enclosing land, and ‘expelling a resident population to create a landless proletariats, and then releasing the land into the privatized mainstream of capital accumulation’ ([14], cited in Benjaminsen and Bryceson [13], p. 336). In this sense, neo-liberalizing nature is characterized by emphasizing the accumulation-led protection, that is, the ultimate goal of protection is to achieve capital accumulation. 

Within the wider discourse of neoliberalizing nature in developing context, the appropriation of land for environmental ends has been described by some as ‘green grabbing’ [40]. It is a process where capital accumulation is achieved through the physical enclosure of ecological space and the transfer of ownership, user rights and control over resources that were previously publicly or privately owned. For some [41,42], green grabbing is characterized by complex processes of deregulation and reregulation and changing patterns of spatial demarcation and control over nature. In places where an urban government does not control initial land ownership and use rights of these reserves, ecological protection enclosures for the purpose of environmental protection have provided legitimacy for the urban government to achieve land usership and ownership. The designation of protected areas means the dispossession of private owners by appropriation, delegitimizing claims through legislation, or dispossession ‘through the market’ whereby those who have valuable assets but are earning incomes too low to permit social growth inevitably have to sell their assets.

To serve the aim of urban growth, land disposition is not the end, which will usually be followed by the clear privatization of state-owned or communally owned social or environmental property. Territory has been made available to investors in various ways, including [11]: (1) the delegation of management authority over protected areas to ‘any person qualified to exercise these powers’, (2) the subdivision of collectively held land to allow for its purchase by private investors and conservation NGOs, (3) the presentation of collective legal titles to rural communities allowing them to enter into business ventures with investors, and (4) the creation of opportunities to make state-controlled territories available to investors through rents and concessions.

Thereafter, the nature of ecological conservation planning and implementation is ‘urban-growth-oriented green grabbing’. This analytical framework is applied in the following case study on the DP in Shenzhen, China.

## 3. Materials and Methods

**Figure 2:**
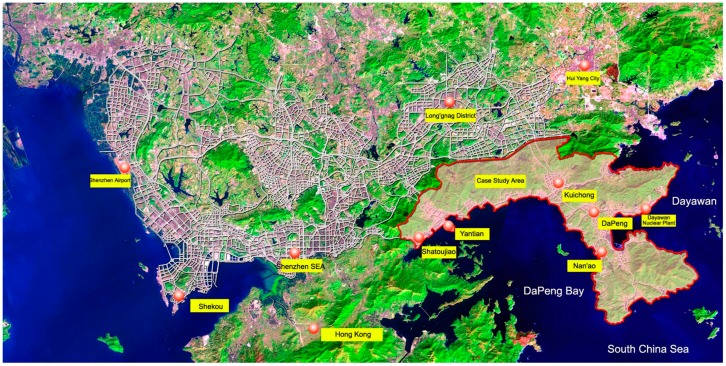
The Geographical Location of DaPeng Peninsula. (Source: The Development Plan of the Eastern Coastal Area of Shenzhen [43,44]).

This research was based on a single case study of the city of Shenzhen. Located southeast of Shenzhen City, the DP is the largest ecologically sensitive area preserved in the city. The region is 296 km^2^ with a total coastline of 133.22 km. The Peninsula is comprised of three administrative units: KuiChong, DaPeng and Nan’ao (Figure 2). Since 2010, the peninsula was designated as the DaPeng District and was under the direct administrative control of the City Council. The subtropical maritime climate nurtures a considerable number of rare plants and vegetables, covering approximately 76% of the peninsula (Table 1).

The study draws upon a continuous monitoring program (since 2008) of Shenzhen’s planning activities. Multiple sources of evidence were involved in the continuous and interactive processes of monitoring. These are policy documentation, non-participant observations, media reports and interviews, all of which was collected and triangulated to ensure their validity and reliability [46].

The first stage of empirical study was contextual study, the aim of which was to map the material, institutional, regulatory, and political context of the case study area and further develop the research framework. Research methods involved include a review of policy documents and open-ended interviews. The documentary analysis is based on documents recording 30 years of the development of Shenzhen since 1979, including strategic plans for Shenzhen from the past 30 years and the strategic plans of this city for the following 20 years, its economic development strategy and social development strategy, etc., and official (e.g., governmental policies) and non-official documents (e.g., newspapers, and memoirs) that describe the evolving process of ecological conservation in the city.

Based on the general findings of the first stage, the aim of the second stage of empirical research was to collect detailed data on specific issues related to conservation on the DaPeng. In line with the research questions as pointed out in the introduction, two main methods were used in collecting data: interview and non-participant observation, together with consistent field notes writing. In total, 34 interviews were undertaken, covering a wide range of participants in the local state regulatory functionaries, national and local planners, ENGOs, local residents etc. All these interviews, including semi-structured interviews and group interviews, were running more than one hour.

Data analysis is a continuous process, which starts during data collection and consistently shapes the ongoing data collection [47]. Three ongoing and cyclical steps are involved and interwoven in data analysis: familiarizing, organizing and coding data; generating categories and subcategories; and modelling relations [48]. The data were first coded and organized to establish a preliminary analytical framework. Those data that fell outside the framework were coded with descriptive free codes for further analysis. This was followed by codes being categorized and sub-categorized through systematic and constant comparison [48]. Informed by the analytical and theoretical ideas developed during the research, categories were further refined and grouped together by revisiting the research questions [48] to model relations among themes. In this sense, a framework was further developed based on the initial framework and formulated according to the theory and the data collected from the field (Table 2). The section followed is organised to address research questions in relation to the proposing and implementation of ecological conservation planning in the case study. The implication of the case study for emerging practices of ecological conservation in urban China, and how the Chinese case contributes to broader theory are addressed subsequently in conclusions.

## 4. Results

### 4.1. Urban Growth and the Politics of Environmental-Ecological Policy in Urban China

The increasing salience of China’s environmental governance has been explained by some using the concept of eco-state restructuring [49]. China’s eco-state restructuring started since the early 1990s, when the deteriorating ecological environment has exerted great challenges towards the rapid economic expansion national wide. Partially influenced by international environmental protection regime, China began to explore the institutional path of sustainable development and the establishment of ecological civilization. In 1992, China announced Ten Strategic Policies for Environment and Development, framing its first national sustainable development agenda. In the 9th Five-Year Plan (1996–2000) ‘sustainable society’ was set as the national environmental goal, seeking to restructure China into a sustainable, mature consumer society, with a resource efficient industrial economy and eco-friendly urbanization.

The goal of ‘sustainable society’ has been explicitly restated in each subsequent Five-Year Plan until 2007, when the theory of ‘eco-civilization’ was set as a national development guideline in the 17th national congress. This new ideology set energy conservation, emission reduction and ecological compensation at the core of its ideological construction, seeking to restructuring the national economy-environment relation from ‘primary-secondary’, ’sequential’ and ‘unilateral’ to ’equal emphasis’, ‘Synchronization’ and ‘comprehensive’ relations, reflecting the adjustment of the state ruling towards sustainable development [50]. 

Since the 18th National Congress of the Communist Party of China in 2013, the institutional system of ecological civilization has been further improved and practiced, with an emphasis on setting CO_2_ emission targets. The core contents of ecological civilization include eight institutional systems, they are: property rights system of natural resources, national space development and protection system, spatial planning system, resource management and comprehensive conservation system, resource utilization system and ecological compensation system, environmental governance system, environmental governance and ecological protection market system, evaluation of ecological civilization performance appraisal and accountability system. These eight systems constituted an ecological civilization system which is of clear ownership, with public participation, both incentive and restraint.

Eco-state restructuring represents a stronger intervention from the state in the field of environmental protection, which is mainly reflected in the following four aspects: Firstly, the strengthened regulation and restriction on the use of space and resources, which is delivered by the strengthened land-use and the spatial planning regulation, the total amount of resources management and the significantly increased national fiscal expenditure on environmental protection. Secondly, the institutionalization of a market-based ecological protection system at the national policy level, including the system of property rights of natural resources assets, resource utilization system, ecological compensation system, environmental management and ecological conservation market system. Thirdly, the establishment of an environmental governance system based on multiple stakeholders’ participation. Fourthly, the setting-up of an ecological performance appraisal and accountability system.

For all cities and regions in China, the Chinese eco-state restructuring has altered the strategic context for urban and regional management, which means both challenges and opportunities. Cities and regions are required to comply with supply-related regulatory targets in relevant laws and legislation. Sub-national governments are under pressure to invest in green communities and green infrastructure; evaluation and accountability for ecological civilization performance means that urban and regional governments need to manage local ecological protection budgets under the goal of ecological civilization development set by the government at a higher level so as to deal with the higher level governments’ penalties (or financial incentives for ecological protection) for failing to meet the targets. On the other hand, participating in ecological protection of market transactions provides local governments new opportunities to offset higher financial returns on conservation input, or obtain economic returns.

Within the context of eco-state restructuring, three kinds of local government governance strategy options have emerged in China. First, some localities engage in environmental protectionism by restricting in-migration, developing local energy solutions and resisting incorporation into wider city-regional or regional networks. Some localities try to reform and adjust the existing growth trajectory to enhance the “greening rate” of industries and reduce the negative environmental externality. There are also some cities and regions that have chosen a third strategy, that is, using low-carbon opportunities (including local carbon tax revenue) to carry out governance reforms to pursue non-growth, de-growth, low consumption or alternative growth strategies. For instance, since 2000, some cities which are able are seeking to move to a higher-road of economic development—a second stage growth model. This growth model focuses on the upgrading of local economies around knowledge, technology and skilled workers (e.g., [51,52,53]). This vision in turn embodies, reflects and creates demands for increased levels of environmental protection, including the protection of environmental amenities in order to attract and retain a burgeoning urban middle class. In Shanghai, for instances, urban regeneration policies emphasizing city beautification and modernization are explicitly promoted by urban governments as a blueprint for a civilized city life [54]. This is because the quality of life and environmental urban amenities serve as one of the key factors in attracting investment and high quality human resources. Zhang et al.’s case demonstrates how housing developers embrace the application of certain types of green strategies to help contribute to building up their competitive advantages in reputation gaining, reduction in construction and operation cost, receiving favourable land prices, and more channels available for financing [55].

### 4.2. The Entrepreneurial City Management of Shenzhen and the Conservation of the Ecology of the DP

Shenzhen is a city located in the South Guangdong Province of China bordering Hong Kong. It is one of the nine special economic zones in the Pearl River Delta (PRD), which has been one of the main areas for economic development in the post-1979 era and a major manufacturing centre. Since its inception as a ‘special economic zone’ (SSEZ) in 1979, the city has grown at an amazing speed from a tiny rural town to a large city in China.

To a large extent, as argued by some [56,57], Shenzhen, in its early years, typified the Chinese entrepreneurial city management model with the city being locked into a growth paradigm in terms of attracting inward investment, increasing local financial revenue and promoting city competitiveness. Due to its geographic proximity to Hong Kong, Shenzhen was defined as the first test field for the Chinese central state [58]. The central state’s intention constituted a strategic context for the policy making of the city government to promote urban economic growth by taking over part of the modular production processes in world manufacturing. Policies were formulated to support the development of comprehensive industries, covering the secondary sectors of the economy, such as trading and commerce, agriculture, stock raising, housing development and urban tourism [58]. This leads to urbanization being dependent on the steady supply of natural resources, including fresh water, fuel, land, food and all raw materials. The urban expansion was further strengthened by the land market that was introduced in the SSEZ in 1987 as a practice to increase the government’s revenue [59]. In this context, Shenzhen’s economy had maintained a double-digit growth rate per year for the two decades since 1980 [60], and the value-added secondary sector of the economy has increased by 86.9% per year during this period with construction and the processing and assembling of industrial products as the leading industries [60].

The 2000s witnessed an increased emphasis within the city on moving away from production and distribution towards tertiary activities and the increased demands of the city to retain its role as a strong player in the intercity competition over command and information functions. This included plans to shift from resource intensive manufacturing towards a knowledge, technology and culture-based economy, entailing a greater emphasis on environmental protection and quality of life than was the case previously [58,59,61,62]. What this ‘high road’ economic development strategy [63] entailed was a greater emphasis on environmental protection and quality of life as one of the key factors in attracting investment and high quality human resources to support a knowledge- and innovation-based economy. Considerable emphasis was placed on the development of tourism across Shenzhen by the city government in support of industrial development citywide.

Our interview with one of the local officials further uncovered the intention of local decision-makers to seek a new method of economic growth to enhance the intercity competitiveness of the city:

Many of the judgments we made were based on the analysis of value. It is about the so-called ‘rationale’. The macro background is the priority of the national ideology - which happens first, the so-called economic development or the ‘harmonious society’, or the scientific development. Starting from this, when the development of one area is concerned, our understanding is that it may not necessarily be the case that we could only achieve economic growth by using up all the land and natural resources, or social resources, it is also something about the effectiveness of land use...The issue is about how to find a more compact mode of spatial development than we previously chose.

(Interview, urban planning and design planner no. 4)

Resources in the DP guarantee and enable the city to supply its economic growth and thus enhance the city’s competitive position under conditions of resource constraints:

‘…when we were valuing the natural resources in the DaPeng Peninsula, we were not treating it as isolated ecological resource. Rather, we took a regional perspective. The DaPeng Peninsula is one part of the regional ecology. The eco-protected area to the northeast of Hong Kong, the whole DaPeng Bay, and the DaPeng Peninsula…these three constructed a regional eco-coastal area. In this area, we have turtle reserves, the volcanic Geopark, and we have also the natural reserves in Hong Kong…so, we are valuing the natural resources of this peninsula at the regional scale. Its value lies in the population it is serving: in the Pearl River Delta region, which is intensively populated, such ecological resources are very rare. To some extent I dare say the value of the ecological resources in the DaPeng Peninsula is much higher than those of the National Reserves in East China…’

(Interview, urban planning and design institutions planner no. 3)

To local governmental officials, nature has not been understood as having a largely external existence; it has been regarded as something that could be materially internalized by certain groups of capital [64]. Government officials assumed that through increased access to resources, nature may have more value to the stakeholders, which on one hand could be the driving force for conservation and on the other hand could contribute to the wellbeing of the local people [2,65]. The increased official recognition of ecological values in the development of the DP by the Shenzhen city government was integrated into a citywide call for attention to environmental protection and ecological regulation. Within this context, the two ecological protection plans of the DP assist achieving urban growth goals by setting up the system of ecological zoning and graded protection areas. In the next section, I will demonstrate and analyse how the urban growth goals were achieved through spatial accumulation with the use of ecological planning.

#### 4.2.1. Reregulation: Land Transfer and the Conservation Plan on the DP

The history of the DP is closely linked with the origin of Shenzhen [66]. For a long time prior to 1949, local residents in the DP relied mainly on traditional fishing and farming. With the establishment of the People’s Republic of China, the DP transitioned from an area where development was guided by traditional ‘fengshui philosophy’ to an area controlled by the ideology of the ‘great leap forward’, which stressed the exploitation of natural resources and the reorganization of nature. When the national reform and openness policy was introduced in the late 1970s, a rural revolution was initiated spontaneously by peasants. ‘By the end of 1980, the contract responsibility system was fully implemented in Bao’an County. One year later, land was cultivated by farmers who had the land use right’ [67]. Under the household responsibility system (HRS), collectively owned woodlots had been distributed to peasants under a variety of contractual forms that granted them an entitlement to the forested land and its harvest [67].

In 1990, the jurisdiction of the Shenzhen municipal local authority was rescaled as a city region, which consisted of the SSEZ (including Luohu, Futian, and Nanshan District) and one county (Bao’an County). The peninsula was by then under the jurisdiction of Bao’an County. According to the law of Land Administration of the People’s Republic of China [68], the land ownership of the DP, except as otherwise provided for by the state, belonged to the county collective.

For the Shenzhen city government, which aims to integrate the DP into the city’s overall development blueprint, the greatest challenge is to have the full possession of the land ownership rights. In other words, collective land needs to be transferred to state ownership. Without such a shift in land ownership, the Shenzhen city government would have no legitimate right to control and distribute space and resources on the DP.

In taking over the land, the city government had two choices: one choice was to acquire the land ownership through land acquisition; another choice was to use the land nationalization policy. The municipal government eventually chose to use the land nationalization policy, which was achieved by transforming the status of Hukou registration of local peasants from ‘agriculture’ to ‘non-agriculture’ on the basis of two laws. One law was the Regulations for the Implementation of the Land Administration Law of the People’s Republic of China [69], according to which land requisition is the only legal approach to changing land ownership. This law, however, also states that when ‘all the members (villagers) of the rural collective organization become civilians’ then ‘land that used to be owned by all the members of the collective will be owned by the state’. This rule, which defines the relationship between land requisition and the local residents’ identity, was interpreted by the municipal decision makers in a reverse way. In the local law Land Urbanization Administration Rules in Bao’an and Longgang, Shenzhen [70], the Shenzhen city government proclaimed the implementation of land transition in Bao’an and Long’gang. Land transition means ‘changes in land nature achieved by changing residents’ identification from rural peasants to civilians’. With the transition of the status of Hukou registration of local peasants from ‘agriculture’ to ‘non-agriculture’, the collectively owned land transformed into state-owned land. In comparison with land requisition, the land nationalization policy is a cost-effective way of obtaining land ownership. It avoids the complex legal procedures required in land requisition to change the nature of land use.

The transfer of a significant quantity of land rights, however, led to tensions between local residents and the regulatory bodies, with a particular issue over compensation. Local standards of money compensation in land transfer differentiated with the national standards, which seemed highly problematic to local residents. Moreover, compensation in the form of land in other places could be only used in developing collective economy and the construction of public infrastructure, which means the deprivation of local residents’ right in maintaining economic development in the long run, including development relied on agricultural production with the transforming of their Hukou registration from ‘agriculture’ to ‘non-agriculture’:

‘In terms of the peasants, land is their life-giver. No matter what happened, for instance, I may be wealthy at present but what if all the money was unstrained expended by my children. Even though since I have the land, I can still survive in a long-term. However, the land nationalization means that now I am compensated by the government, but I have to take care of my own life from now on…’

(Interview, Local resident no. 3)

The landownership transition from the collective to the state also means that local communities would lose their legitimacy and flexibility in intervening into the development of Town and Village Enterprises since the provision of land and factories was no longer promised. In the DP, the collective economy is developed in the form of Community Corporation, in which registered local residents are shareholders. The chairman of the board is the Party Secretary of each community, whom is recommended by the higher level of government, and then further voted by villagers (Interview, Long’gang District Government official no. 2). There were three steps for these Community Corporations to be involved in productive activities: ‘to stimulate investment’—‘to provide land or factories’—‘to obtain rents from land and house letting’ (Interview, Local environmental activist no. 2, 2009). The counties and villages were in charge of building factories, while local residents, in turn, became the clipping coupons class who lives on land rents and shares collective bonus. However, the land transfer from the collective to the state represents the end of the right of the collective in the use and allocation of land and resources.

**Figure 3:**
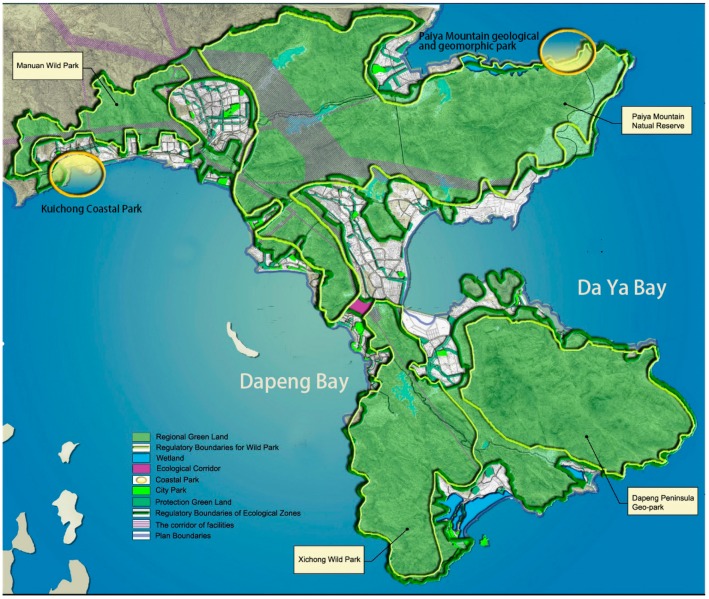
Protected areas in the Shenzhen Eastern Ecological Group Plan (2005–2020). (Source: Author’s compilation based on the Shenzhen Eastern Ecological Group Plan (2005–2020) [43,44]).

Unsurprisingly, anger about the insufficient compensation leaded to fierce resistance towards land transfer. One of the major strategies for the local residents to protest against the governmental regulation is illegal constructions. Within this context, in November 2003, the Urban Planning & Design Institute of Shenzhen (UPDIS) was commissioned by the Shenzhen Planning and Land Bureau to develop the Shenzhen Eastern Ecological Group Plan (2005–2020). Approximately 230.17 km^2^ of ‘regional green lands’ were designated for protection in the DP, including agricultural areas, source water protection zones, green belts, tourism resorts, suburban parks, and nature reserves (see Figure 3). The municipal government’s emphasis on environmental destruction on the DP makes this Plan hard to be refused by the local peasants. What is significant here is that the Plan was layered onto the land transfer policy, with the ecological regulation further restricting existing and future social and economic activities of localities. 

#### 4.2.2. Deregulation: Spatial Interest Redistribution through Ecological Territorialization

**Figure 4:**
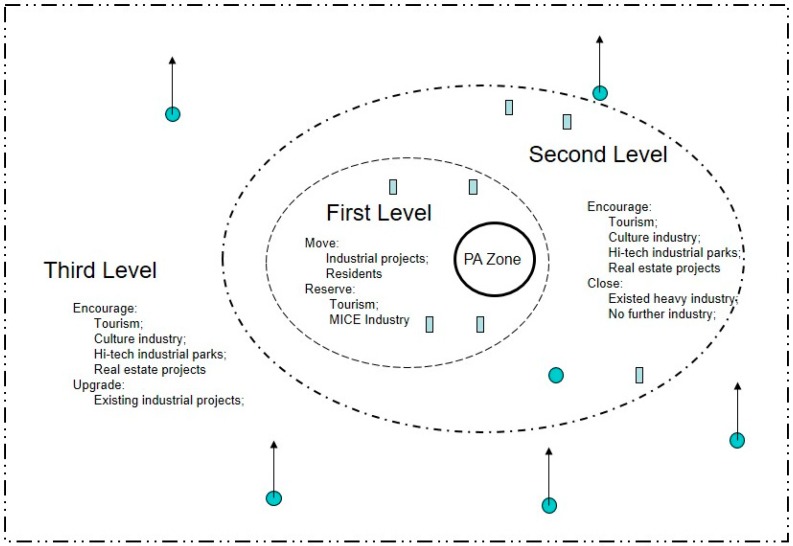
Three levels of construction control (an abstraction).

Once the Shenzhen city government controlled the land ownership of the DP, the government redistributed and re-territorialised the spatial land resources of the DP. In 2007, the DaPeng Peninsula Protection and Development Implementation Strategy (thereafter ‘the Strategy’) [18] was approved. In this Strategy, the concept of ‘graded protection areas’ was employed to regulate and govern space in Shenzhen based on the ideas of ‘core ecological protection zones’(CEPZ) [18] and ‘construction control areas’(CCA) [18]. Two management territories were proposed by the Shenzhen city government in the Strategy—core ecological protection zones and construction control areas—in an attempt to combine development with conservation (Figure 4).

In the following sections, I will further explore how urban spatial accumulation was achieved through the process of ecological territorialisation. For the convenience of discussion, the ecological territorialisation is focused on the redistribution of the CEPZ and the deregulation of CCA.

#### 4.2.3. The Appropriation of Land for Ecological Reserves

The CEPZ in the strategy is mainly the Dapeng Peninsula National Geopark. Geoparks are part of an integrated concept of protection, education and sustainable development [71,72]. Geoparks denote a direct relationship between people and geology and the ability of a site to serve as a focus for economic development, for instance, the creation of innovative local enterprises, small businesses, cottage industries and new jobs that generate new sources of revenue (e.g., geotourism and geoproducts) [72]. To a large extent, the ideology of conservation-with-development that underpinned the Geopark sparked debates on new opportunities for the development of both the whole city and the DaPeng Peninsula based on the protection of ecological areas (Interview, Shenzhen city government official no. 1). For governmental officials, ‘the ecological resource opportunities of the eastern part, in particular, of the DaPeng Peninsula have significant potential for developing a regional and international tourism industry, which will bring a new dimension of promise to Shenzhen that will benefit from new regional cooperation and competition’ [18]. ‘We thought it would be a good project…since we can develop some tourism related business…we could benefit from that if more tourists could be introduced to the area’ (Interview, Long’ gang District Government official no. 1). Most significantly, ‘the success of the application for the DaPeng Peninsula Geopark will be a key project…combined with the construction of the wilderness parks and supported by service infrastructure, these will be the ‘calling card’ for the tourism industry of the DaPeng Peninsula’ [19]. In 2004, the geological society of China put forward a set of recommendations regarding the establishment of a Geopark on the DP peninsula, which soon attracted the attention of the mayor and gained great support from the Shenzhen city government with an application to the Provincial Land and Resource Bureau for the establishment of the DaPeng Peninsula National Geopark in June 2005 [73].

The Shenzhen city government’s protection of the DP Geopark does not follow the way of isolated protection. Instead, the municipal government gives local residents certain economic benefits and sharing power under the concept of ‘development and protection’ represented by the DPK projects. The Shenzhen city government guided indigenous residents on the DP in developing several temporary (in principle, no more than three years) projects named DPK projects in order to obtain substantial benefits from the conservation and management of the DP [43]. Through these DPK projects, indigenous residents are empowered to take greater control of their own lives and secure a better livelihood in the context land ownership that has been transferred from the collective to the state [74].

For the Shenzhen city government, the primary intention for adopting DPK projects was to reconcile the local contradiction between conservation and local development; the supposition was that society-nature contradictions, especially those that emerged during the process of land transfers and the implementation of the ecological zoning, could be resolved by giving a degree of flexibility to local residents. Local residents are thus encouraged to develop tourism by establishing their own cooperation with relatively small investments.

‘If all the lands were tightly protected without any development this sure would arouse strenuous objections from the indigenous residents. In that context, they might just construct blindly because they could immediately see the effects of their construction. However, the results of this will be therefore that we conduct the project of “Duan Ping Kuai”’.

(Interview, Shenzhen city government official no. 3)

There is also the recognition that some flexibility was required to ensure the longer-term success of the policy of ecological regulation. In the context of limited local revenue availability, the DPK projects were regarded as a transitional strategy to ensure the longer-term success of the policy of ecological regulation without ensuring sufficient ecological protection mechanisms at the local scale.

‘We proposed the project of transfer payments, in particular transfer payments for environmental protection, which means giving certain amount of financial subsidy to the indigenous residents as compensations for their pecuniary loss, in order to protect the environment. However, the compensation seemed to be an utterly inadequate measure to meet their demands of economic development. Even if the collective organizations could not admire the development of the western part, the individual desire for being wealthy could hardly be controlled...such a power from the bottom-up pressured us to propose the project of “Duan Ping Kuai”’.

(Interview, Shenzhen city government official no. 1)

The DPK project could also be regarded as the tendency of the Shenzhen city government to use ‘neoliberal environmental measures to solve problems arising within…the wider economy and society’ [11]. For instance, most of the DPK projects (27 in total) are run by communities themselves and developers, covering residential districts, leisure and tourism infrastructures and so on [43]. ‘By providing limited access to certain resources, investing in alternative income-generating activities’ ([2], p. 8), for instance, by allowing resources to be privatised and marketised, it was argued that the local people may be compensated for and obtain the power over productive assets to a limited extent. However, this kind of revenue sharing has benefited interest groups beyond the local area rather than local parties (Table 3).

As demonstrated in the case of the Bantianyun Coastal Leisure and Tourism Facility Project, the ‘community’ may be diverse in a number of ways, e.g., economic status, gender, social and cultural differences [2,75], and the close relations between local social actors and outside agencies may influence resource use and management at the local level [76]. Located within the CEPZ, Bantianyun Coastal Leisure and Tourism Facility is one of the DPK projects. In many respects this project did not follow the original official plan. The building process lasted for two years from 2005 to 2007 with the total land use scale of 2468.89 m^2^, including 900 m^2^ existing construction area which is 100 m^2^ over the required and approved scale. Though a total 5,000,000 Yuan was collected by the community to be invested in the project, the project is now co-managed by both the Community Cooperation and an enterprise

#### 4.2.4. Territories Available to Industrial Investors

The municipal government has selectively introduced new types of industry through further territorialisation within in the CCA, aiming to support the development and transformation of city’s urban industry. On the premise that the key ecological resources are protected, the ‘construction control areas’ involve areas with three degrees of construction control [19]. Areas under the first level of control are where industrial projects are generally prohibited, yet tourist activities (like marine leisure activities) and Meeting, Incentive, Conference and Event (MICE) industries are allowed. In the second level of construction controlled areas, economic activities are regulated according to their different natures: existing industrial projects and some sea-enclosure aquaculture projects with heavy pollutants are required to be closed or relocated; further industrial constructions are prohibited; yet tourism, culture industry, hi-tech industrial parks and some real estate projects are encouraged. In areas under the third level of control, the economic activities that are prohibited and encouraged are similar to those of the second level of control, though existing industrial projects are encouraged to undertake redevelopment [19]. The introduction of either ecological agriculture or high-tech industries is essentially the re-excavation of values of local natural resources. In this process, the main subjects of the development and utilization of local resources is transformed from the original local residents into new industrial developers.

The Shenzhen city government had the intention to develop eco-agriculture and modern oceanic industries in the DP. As noted in the eleventh five-year plan of Shenzhen [77], ‘the DaPeng hi-tech agriculture park and the exemplary hi-tech biologic district of the eastern ocean were based on high planning and construction standards’ and were expected to be constructed in the DP [77]. Resources were valued for their potential contribution to facilitating the development of service industries, including a culture industry and especially a sports industry; tourism industries, including business holidays, eco-recreation, coastal recreation (geological parks and mountain adventures), and the utilization of the cultural value of the resources; and the exhibition industry as one of the new industries to utilize the city’s modern services. 

Industries, for instance, the high-tech manufacturing industry, new materials industry, and fine chemistry, are also expected to be developed in the DP in order to upgrade the structure of the hi-tech industry and advance manufacturing and to provide new opportunities for the economic development of the city.

‘At that time the Secretary of the Municipal Party Committee proposed the idea of “appropriate development of manufacturing industry”…the aim of constructing the fine chemistry industry in Baguang was to enter into the industrial supply chain of petroleum industry in Daya Bay. There has been construction in Baguang before, so it is one of the industrial development policy areas…’

(Interview, Shenzhen city government official no. 3)

The Baguang fine chemical industry project is a case in point. Located in the DP, Baguang Village consists of 18 residential groups with a population of 2380 scattered along a 16 km coastline [78]. The 1.5 hectares of the *Heritiera littoralis* Dryand is found to be the best preserved natural silver-leaf tree community in China [79]. In 2005, the area was listed as a national nature reserve for ancient *Heritiera littoralis* [80]. However, along with this recognition of the ecological value of Baguang came the city council’s decision to construct a fine chemical industry park in the same area. In fact, this area has been surrounded by various industrial projects. The Fine Chemistry Project started in July 2003, and the city government’s expectation was to have had an opportunity to bring in significant local revenue. In 2005, the research on the plan for the Shenzhen Fine Chemistry Industrial Park undertaken by the China Petroleum Chemistry Industry Plan was approved by the Shenzhen City Council [81]. This project was integrated into the Guangdong Province Eleventh Five-Year Plan for the construction and planning of five major petroleum sites. The industrial outputs of this project were expected to be approximately 1200 billion Yuan per year [82].

In fact, (the government) expected to introduce Shell in 1992 or 1993 when the DaYa Bay was under construction. Due to certain reasons, the sort of macro adjustment, it was not successful. In 2003, the Shell project was successfully introduced. In this sense, the Mayor made the decision to construct this project, which is to let Baguang to take over its downstream products.

(Interview, urban planning and design planner no. 6)

In addition, 7.5 km of artificial coast line is needed for the development of the Fine Industrial Park through 10 km^2^ sea landfill. In this sense, the whole residents of the Baguang, which accounts to 1492 household (which is 91% of the total 1631), were required to move off to Kuifeng Community in Kuichong Street [78,83]. Even though this proposal was cancelled due to pressure for ecological conservation from various groups and intensive media coverage in 2011, a new development plan was proposed by the Shenzhen city government in 2013 to develop Baguang as an international biological valley constructing biological industrial structures to increase international competitiveness.

## 5. Discussion and Conclusions

The strategic and spatial selectivity of the plans and the resulting struggles in their implementation across the DP reveal the logic of ecological protection in the context of China’s urban political economy. The new characteristics of Shenzhen’s city politics highly reflect the glocalization and entrepreneurship of city governance and new global-local environmental politics constructed around ecological modernization and the partial greening of capital [84,85]. Ecological conservation planning on the DP is a form of ‘urban growth oriented’ conservation where the ecological value of the DP has been highlighted by the growth regime as a new resource to support city growth. Ecological space protection has become the main practice to accomplish urban spatial and capital accumulation through resource reorganization and the redistribution of interests. DaPeng Peninsula’s ecological spatial protection has gone through processes of both reregulation and deregulation. The first stage was the exclusive process of enclosure through the designation of ecological reserves; and the second stage was deregulation, which was achieved through the redistribution of spatial benefits among interest groups within the city. The above process comprises the strategy of green grabbing, which also raises the question of environmental injustice, where land ownership and the benefits of development has been transferred away from the local residents to new interest groups.

Ecological conservation in China has similarities with other research on neo-liberal nature, yet it still has its’ own characteristics. Compared to most studies that discuss ecological protection practices in protected areas that are far from cities in developing countries, it is impossible to discuss ecological conservation without an understanding of urban development politics in China. Under the background of decentralized governance in China [86], cities are not only engines of economic development but also the main subjects of implementing ecological protection. Therefore, the politics of urban development is an important background and construct that cannot be ignored in understanding ecological conservation. In this context, ecological conservation in rural areas is represented by what I have termed ‘urban-growth-oriented green grabbing’ in this paper, which has several features as follows.

At the outset, urban growth comprises the main motivation of city governments to be involved in ecological conservation practices in rural areas. One of the particular contributions of the paper has been to highlight ways in which ecological conservation practices might be used by city governments to serve interests in social and spatial restructuring, and this provides further evidence that the designation of protected areas needs to be placed within the wider politics of and urban development [39]. It has been seen that the designation of ecological reserves in the DP is part of broader strategies to reorder state space serving socio-political as well as ecological ends. By taking a ‘non-Western’ perspective, the paper does not necessarily fit with conceptions of nature conservation based on a hierarchy of designations imposed top-down in a strong nature-first paradigm [87]. Rather, the paper maps out a slightly different dynamics of ecological urban politics based around the strategies for regulating growth. As in the case of the DP, in the context of the emerging new phase of ecology-based development, the city of Shenzhen appears to have an increasing need for ecological conservation in order to retain its role as a strong player in intercity competition with respect to its command and information functions. Ecological conservation in the DP was not necessarily just about securing a ‘biophysical fix’ in response to pressures and demands of nature conservation, but reflected the use of ecological arguments to help support a re-regulation of society and space as the city moved to a new phase of development. In this sense, what Harvey has termed ‘accumulation through dispossession’ directly points to accumulation for urban growth through spatial accumulation. Second, a key step in supporting urban growth has been green grabbing from the rural. The role of green grabbing in urban growth is manifested in at least three aspects:

(1) the case of the DP echoes with Chen’s argument that China’s urban space accumulation strategy requires the intervention of the state power, which is the key to territorialisation and land commercialization. The particularity of China’s background lies in the fact that in the context of the Chinese transitional economy, the involvement of state power has played an important role in converting rural land into the land owned by the city, beyond the use of states transforms previously untradeable things into tradable commodities [88] through re-distributing spatial resources which are previously controlled by collective communities.
(2) Green grabbing has created the necessary legitimacy for the intervention of the state power, where the demand for space in supporting urban growth is difficult to be fulfilled through the direct intervention of the state power, given the increasingly severe socio-economic conflicts. What Chen’s discussion did not explain is the special role played by the enclosure of space for the purpose of ecological protection within the state intervention. In China, the urban-rural dichotomy poses new challenges for urban growth. The core of these challenges is the differences in land ownership between urban and rural areas, which provides an opportunity for the emergence of green grabbing. Theoretically, the green grabbing process involves two steps: first, obtaining ecological resources in the name of ecological conservation, followed by transforming ecological resources into the basis of capital accumulation. In contrast to the earlier mandatory nationalization of land, the ecological conservation targets contained in green grabbing provide sufficient legitimacy for state involvement and provide an opportunity to mitigate possible conflicts that arise during the process of protection. In the case of the DP, for instance, the promulgation of policies in designating geological parks and conducting ecological conservation planning laid the foundation for the state to obtain land ownership in the name of ‘conservation’, which thus soften conflicts arising from the process of land nationalization.
(3) Similar to other research in green grabbing (e.g., [13]), in the case of the DP, the city’s accumulation strategy is achieved through the process of green grabbing in terms of the reregulation and deregulation of nature reserves. That is, to obtain space accumulation by dissociate the original users away from claiming their rights to the land and properties, and further deregulating and territorialisation through conservation strategy to facilitate capital accumulation by some powerful actors. Such an accumulation process, however, is not simply based on the primary and secondary land development [89]. The process of deregulation led by green grabbing emphasizes a new way of excavating the value of ecological resources, which creates a new condition for capital accumulation. One of the manifestations is the delineation of geo-park. The city government pays more attention to the indirect use value of geo-park in the development of urban tourism and the creation of urban reputation. During the process, by transforming the original collectively-owned entity into a state-owned entity, the original collective-owned landowner migrates to other parts of the city, and the city government gains the space and benefits of tourism development [13]. The second way of urban accumulation is the reconstruction of natural resources. Following the Marxist tradition, Neil Smith argued that nature has been produced and internalized by modern capitalism as strategy of profitability, which is, according to Boyd etc. [64], the real subsumption of nature to alter its biophysical properties so that it offers enhanced possibilities for capital accumulation. For instance, gen-technology in making some genetically modified organisms [11]. In the case of the DP, local governments tried to re-excavate nature through the development of new types of industries in order to carry out urban capital accumulation.

Third, the results of the urban-growth-oriented green grabbing is a new type of deprivation. The traditional urban-rural binary in China emphasizes the possession and development of rural land by cities under the traditional development model [90], where the value of rural ecological resources is mainly based on the excavation of its direct use value. The new urban-rural relationship is representing a hidden possession-development relationship, where the basis of urban development has shifted from the in-depth use of the direct use value to indirect use value of ecological resources. In this urban-rural relationship, rural natural ecological resources have become a major factor in winning competition among cities. As a result, the protection of rural land is associated with the commercialization of natural capital. Although the traditional urban-rural duality was replaced by new urban-rural relations, environmental injustices committed between these two still remains. It is noteworthy that this urban-rural relationship is still an unequal urban-rural relationship. The original occupants and users of rural natural ecological resources are excluded and are replaced by new entities in the process of the redistribution of land spatial resources. As in the case of the DP, even though the government within the core ecological reserve hopes to create opportunities for local residents to share economic income through the development of the DPK projects, most of the benefits are still obtained by private investors outside the local area. The introduction of new types of industries has also shifted future developmental benefits from local residents to industrial sectors. In this sense, the local residents are not only deprived of the land, but also their local culture and possible future development opportunities. Eventually, the results of the urban-growth-oriented green grabbing have been what Dooling has called ecological gentrification [32].

In a wider context, there are two main contributions from this article. First, this article attempts to shed new light on neoliberal conservation discussions by introducing the concept of “urban-growth-oriented green grabbing” while addressing the urban scale. Second, this research reflects the necessity of bringing conflicts arising from the process of the neoliberalization of rural ecological resources into the discussion of urban growth politics from the perspective of urban-rural interactions.

## Figures and Tables

**Table 1:** Significant geological and ecological resources on the DP. (Source: author—compiled based on [18,45]). Italicize species names.

**Plants and vegetation**	*Antirhea chinensis* (Vulnerable) *Amentotaxus aragotaenia* (V) *Brainea insignis* (V) (which has been listed in the CITES) *Camellia grathamiana* (Endangered) *C. sinensis* var.assamica (V) *Castanopsis kawakamii* (V) *Ceratopteris thalicroides* (V) *Cinnamomum camphora*(V) *Drmacarpu longun* (V) *Glehnia littoralis* (V) *Gymnosphaera podophylla* (V) *Ixonanthes chinensis* (V) *Nauclea officinalis* (Rare)	*Comm.Syzygium odoratum* *Comm.Machilus chekiangensis+Schima superba* *Comm.Canariumpimela+Chrysophyllum roxburghii+Antidesmabunius* *Comm.Schefflera octophylla-Gordonia axillaris* *Comm.Castanopsisfargesii+Castanopsis fabri)* *Comm.Ficusfistulosa+Mallotushookerianus+Sterculialanceolata)* *Comm.Kandeliacandel+Aegicerascorniculatum-Acanthus ilicifolius*
**Geological relics**	The geological features of ancient volcanoes The ancient volcanic lithofacies Stratigraphic section Palaeontology fossils Canyons river erosion landform types Coastal caves Marine accumulation land-form Fault tectonic geomorphology Prehistoric cultural relics	
**Coastlines**	14 Coastline sections	

**Table 2:** How research questions lead to conceptual generalization of planning activities in the DP.

Research Questions		Conceptual Generalization		Planning Activities	Key Stakeholders Involved
Why and how ‘wild’ or ‘natural’ areas are captured by and incorporated into urban space, and how land appropriation for urban ecological amenities forms a new model of appropriation in China?	Why these two ecological conservation plans emerged in the DP Shenzhen?	Urban growth	The entrepreneurial city management of Shenzhen	Considerable emphasis was placed on the development of tourism across Shenzhen by the city government in support of industrial development citywide.	Shenzhen City Government; Shenzhen City Planning Bureau
	In what ways ecological conservation plans are implemented in the case study area?	Green grabbing	Spatial accumulation through Reregulation	Land transfer	Shenzhen City Government; Local residents
				The proposing of conservation plans on the DP	Long’gang District Government; Shenzhen City Planning Bureau
			Capital accumulation through deregulation	The appropriation of land for ecological reserves	Shenzhen City Government; Dapeng Peninsula National Geopark Administration; Local residents; Real estate developers; DPK projects operators in the DP
				Territories available to industrial investors	Shenzhen City Government; Industrial investors

**Table 3:** Details of selected DPK projects. Source: Author’s compilation based on [43].

Project	Land Ownership	The Type of Construction	Land Area (m^2^)	The Size of Construction (m^2^)	Collective Income (10,000 Yuan)	Bonus Share for Local Residents per Person per year (Yuan)	Management
DaPeng Suocheng Car Park	Collective land (existing construction); State land (planning construction)	Car park, One-story retail building	7600	820 (for retail), 7000 (for car park)	12	80	Management groups organized by the Community Revenue Cooperation
Dongyu Coastal Tourism Service Centre	Collective land (non-agriculture land)	Port and two-story sea-food retail building (existing construction)	5000	1500	25	800	Management groups organized by the Community Revenue Cooperation
Guanyin Mountain Service Restaurants	unclear	Two-story restaurant and retail building	3000	1780	32 (estimated)	500 (estimated)	Rent out to be managed by enterprises
Nuclear Plant Dormitory	Collective land (19,731.5 m^2^) State land (768.5 m^2^)	Three dormitories (three or five-story)	20,500	25,000	36 (estimated)	240 (estimated)	Community Revenue Cooperation
Bantianyun Coastal Leisure and Tourism Facility project	Unclear	Three Holiday Inns (two-story wood terrace), corridor, car park and service infrastructure	2500	900	50	2000	Co-managed by the Community Revenue Cooperation and enterprises
Egong Wan ‘Eco-tourism Park	Unclear	Sixteen Holiday Inns (two-story wood terrace), one restaurant, one Sauna building, one cafe, corridor, port, car park and service infrastructure	6000	2280	100	600	Co-managed by Community Revenue Cooperation and enterprises
